# Use and effectiveness of tocilizumab among patients with rheumatoid arthritis: an observational study from the British Society for Rheumatology Biologics Register for rheumatoid arthritis

**DOI:** 10.1007/s10067-016-3485-5

**Published:** 2016-12-02

**Authors:** Mari Kihara, Rebecca Davies, Lianne Kearsley-Fleet, Kath D. Watson, Mark Lunt, Deborah P.M. Symmons, Kimme L. Hyrich, on behalf of the British Society for Rheumatology Biologics Register

**Affiliations:** 1 Arthritis Research UK Centre for Epidemiology, Division of Musculoskeletal and Dermatological Sciences, School of Biological Sciences, Faculty of Biology, Medicine and Health, The University of Manchester, Manchester Academic Health Science Centre, Room 2.800 Stopford Building, Oxford Road, Manchester, M13 9PT UK; 2 Department of Rheumatology, Tokyo Medical and Dental University, 1-5-45, Yushima, Bunkyo-ku, Tokyo, 113-0034 Japan; 3 NIHR Manchester Musculoskeletal Biomedical Research Unit, Central Manchester University Hospitals NHS Foundation Trust and University of Manchester Partnership, Oxford Road, Manchester, M13 9WL UK

**Keywords:** Biological disease modifying anti-rheumatic drugs (bDMARDs), Observational study, Rheumatoid arthritis, Tocilizumab, Treatment outcome, Treatment selection

## Abstract

The aims of the present study are to describe the characteristics of rheumatoid arthritis (RA) patients selected for tocilizumab (TCZ), compare the “real-world” effectiveness of TCZ and tumour necrosis factor inhibitors (TNFi) when used as a first biologic and assess the influence of past biologic exposure/concurrent methotrexate (MTX) therapy on post-TCZ treatment outcomes. The British Society for Rheumatology Biologics Register (BSRBR-RA) is a prospective cohort study following RA patients starting biologics in the UK. This includes patients starting TCZ as first or subsequent biologic, alongside biologic-naïve patients starting TNFi. Six-month disease activity and 1-year drug survival were compared between biologic-naïve patients starting TCZ versus TNFi and first-line versus subsequent TCZ users and TCZ users with MTX versus without using regression models adjusted by propensity score. Two hundred seventeen patients started TCZ, and 2419 started TNFi as first biologic. Seven hundred seventy-seven started TCZ after other biologics. First-line TCZ users had a higher prevalence of pulmonary fibrosis and cancer history than TNFi users. The first-line TCZ users were more likely to achieve DAS28 remission at 6 months than first-line TNFi, but other improvement markers were similar. The treatment response at 6 months was similar between subsequent-line TCZ users and first-line users after adjusting for baseline patient differences. Concurrent MTX use was not associated with treatment response in either first- or subsequent-line TCZ users. TCZ has been primarily used as subsequent-line biologic in the UK. When used as first line, the response appears similar to that observed in patients starting TNFi, suggesting that clinical response alone should not decide between initial biologic therapies.

**Electronic supplementary material:**

The online version of this article (doi:10.1007/s10067-016-3485-5) contains supplementary material, which is available to authorized users.

## Introduction

Cytokines such as tumour necrosis factor (TNF)-α, interleukin (IL)-1 and IL-6 play a fundamental role in the pathogenesis of rheumatoid arthritis (RA) [1]. Biological disease-modifying anti-rheumatic drugs (bDMARDs) targeting these cytokines have resulted in improved outcomes among patients with RA resistant to traditional synthetic DMARD (sDMARDs). Drugs which inhibit TNF (tumour necrosis factor inhibitors (TNFi)) were the first available biologic therapies [2], but the choice has expanded, and since 2010, tocilizumab (TCZ), a humanised monoclonal antibody against the IL-6 receptor, has been available for use in RA. In the UK, the drug is approved by the National Institute for Health and Care Excellence (NICE) for use both as a first-line biologic therapy after failure of two sDMARDs and as a subsequent-line biologic therapy after failure of TNFi in patients with severe RA (DAS28 >5.1) [3–5].

With an expanding choice of biologic therapies for use in patients resistant to sDMARDs, there is little information on how these therapies are being selected in clinical practice, as well as a lack of data regarding comparative effectiveness between therapies to help clinicians guide choice of therapies. There also has been little evidence from routine care regarding the factors associated with the optimal effectiveness of TCZ. For example, the impact of prior bDMARD exposure history and concomitant use of methotrexate (MTX) on the effectiveness of TCZ have not been assessed sufficiently.

Whilst a randomised controlled trial (RCT) (ADACTA study) suggested the superiority of TCZ monotherapy in reducing DAS28 compared to anti-TNF adalimumab (ADA) monotherapy among bDMARD-naïve patients with inadequate response to MTX [6], there have been no other RCTs comparing the efficacy of TCZ with TNFi directly. Three cohort studies (two retrospective and one prospective) have compared the effectiveness of TCZ and TNFi, which also suggested the superiority of TCZ [7–9]; however, there is a lack of large comparative effectiveness studies in routine clinical settings.

There is also limited or inconsistent information regarding factors associated with better response to TCZ. Recent prospective studies of patients who started TCZ have suggested that TCZ may be more effective for patients when used as a first biologic compared to those patients receiving the drug after failure of other biologics [10–12]. While evidence has suggested that TNFi may be more effective when used in combination with MTX, this is less clear for TCZ. Two clinical trials (the ACT-RAY study and the SURPRISE study) and three cohort studies suggested that concomitant MTX may lead to favourable treatment outcomes [10, 13–16]; however, a retrospective study, a European collaborative study and ACT-LIFE study from routine clinical settings suggested no difference in effectiveness of TCZ when combined with MTX [11, 13, 17, 18]). Limited evidence from routine use is currently available.

Further information regarding the “real-world” effectiveness of TCZ when used at different points in the RA treatment pathway will provide better evidence on which to frame healthcare decisions. The aims of this analysis are therefore to (1) describe characteristics of patients with RA selected for TCZ in the UK, (2) compare the real-world effectiveness of TCZ to TNFi over the short term (first 6 months) when used as a first biologic and (3) assess the influence of concurrent MTX therapy and past biologic exposure on short-term treatment outcomes following TCZ.

## Methods

### Patients

The British Society for Rheumatology Biologics Register for RA (BSRBR-RA) is a national prospective cohort study collecting information on patients aged 16 or older with a physician’s diagnosis of RA starting biologic therapies. This analysis included all either patients registered with the BSRBR-RA starting TCZ (either as their first biologic (first-line TCZ cohort) or those who had already received biologic therapies and were switching to TCZ (subsequent-line TCZ cohort) or starting TNFi therapy (etanercept—Enbrel (ETN), infliximab—Remicade (IFX), adalimumab—Humira (ADA) or certolizumab pegol—Cimzia (CZP)) as their first biologic between 01 January 2010 and 30 November 2015.

### Baseline data

At the start of bDMARD therapy, baseline data were collected from the hospital including patient demographics; smoking status (current smoker/ex- or never smoker); height (cm) and weight (kg) to calculate body mass index (BMI) (kg/m^2^); disease duration (years); past and current anti-rheumatic therapies; rheumatoid factor (RF) status; extra-articular manifestations of RA (EARA) including pulmonary fibrosis, sicca syndrome, serosal involvement (pleuritic/pericarditis), eye involvement, systemic vasculitis and other specified systemic features based on physicians diagnosis; 28 swollen and tender joint counts (SJC, TJC); patients global assessment visual analogue scale (VAS) (0–100 mm) erythrocyte sedimentation rate (ESR) (mm/h); and/or C-reactive protein (CRP) (mg/dl). Ever existence of comorbidities (hypertension, angina, myocardial infarction, stroke, epilepsy, asthma, chronic bronchitis/emphysema, peptic ulcer, liver disease, renal disease, tuberculosis, demyelination, diabetes, hyperthyroidism, depression or cancer) was captured using a tick list. A list of current medications was also obtained, and statin use was used as a surrogate for baseline hyperlipidaemia. In addition, a comprehensive history of prior cancer was obtained through linkage to the UK national cancer register. Patients complete the Health Assessment Questionnaire (HAQ) adapted for UK use [19].

### Follow-up data

Follow-up data were captured from the hospital and patient every 6 months for 3 years and then annually from the hospital only. Hospital data include the most recent DAS28 and changes to anti-rheumatic therapy including stop reasons and adverse events. Patients complete a HAQ.

### Data analysis

This study compared the baseline characteristics and treatment effectiveness at 6 months between the (1) first-line TCZ cohort versus first-line TNFi cohort, (2) first-line TCZ cohort versus subsequent-line TCZ cohort and (3) first-line/subsequent-line TCZ users with and without concomitant MTX. All data available up to 31 May 2016 were included.

Treatment effectiveness at 6 months was described using the following measures: change in DAS28 and its components, European League Against Rheumatism (EULAR) response [20], DAS28 remission [21], change in HAQ score, the proportion of patients who achieved the minimal clinically important difference (MCID) in HAQ, defined as a ≥0.22 decrease in score [22] and the drug retention rates up to year 1. DAS28 was calculated using the ESR where available. However, if only a CRP measurement was provided (27% of patients), the DAS28-CRP [23] was calculated instead (online Supplementary Table 1). Non-parametric descriptive statistics were used to compare baseline characteristics between cohorts. Regression models were used to compare EULAR response (ordinal models) and DAS28 remission (logistic regression) between treatments. Each regression model was adjusted by (1) age and sex and (2) the estimated propensity score using inverse probability of treatment weighting (IPTW) without trimming. [24] The propensity score was determined separately for each comparison. To estimate each propensity score, logistic regression models including factors showing significant differences between cohorts at baseline and the factors that may predict changes in outcome were constructed. Drug survival up to year 1 was described using Kaplan-Meier curves and compared using the log-rank test.

Missing data were accounted for in regression models using multiple imputation, using the mi package in Stata with 20 iterations. Information regarding missing data is included in online Supplementary Table 1. When it was not possible to calculate the DAS28 due to one or more missing components, the DAS28 score itself was imputed. All analyses were performed using Stata (StataCorp 2013, Stata Statistical Software: Release 13, College Station, TX: StataCorp LP).

## Results

From 01 January 2010 until 30 November 2015, 2636 patients registered with the BSRBR-RA starting their first biologic (217 TCZ; 2419 TNFi: 884 ETN, 51 IFX, 542 ADA, 942 CZP), and 777 patients registered starting TCZ as a subsequent biologic therapy.

### Comparison of baseline characteristics and treatment effectiveness between patients starting first-line TCZ or first-line TNFi following inadequate response to sDMARDs

In general, there were few differences between patients starting TCZ or TNFi as first-line biologic therapy (Table 1). Noted differences largely reflected background comorbidity: a history of pulmonary fibrosis was higher in the TCZ group compared to TNFi (5 vs 2%, *p* = 0.001), as was prior cancer (9 vs 5%, *p* = 0.001). The proportion of MTX use at baseline was similar between the two cohorts, but the TCZ cohort had a slightly lower percentage of previous MTX use (94 vs 96%, *p* = 0.05). The TCZ cohort had lower VAS score (72 vs 78 mm, *p* = 0.002), but the disease activity according to DAS28 was similar to the TNFi cohort (6.2 vs 6.0, *p* = 0.18).

**Table 1:** Baseline characteristics of patients initiating first-line TNFi, first-line TCZ and subsequent-line TCZ cohort

	First-line TNFi (*N* = 2419)	First-line TCZ (*N* = 217)	*P* value^a^	Subsequent-line TCZ (*N* = 777)	*P* value^b^
Demographics					
Age, median (IQR)	57 (48–66)	58 (49–66)	0.7	58 (49–66)	0.77
Female, no. (%)	1833 (76)	159 (73)	0.4	616 (79)	0.06
Current smokers, no. (%)	472 (20)	42 (20)	0.9	93 (19)	0.9
BMI, median (IQR)	27.4 (23.7–32.0)	28.4 (24.7–32.0)	0.08	27.7 (24.0–32.9)	0.4
Disease characteristics					
Disease duration (years), median (IQR)	5 (2–13)	*4* (*2*–*12*)	0.2	*12* (*6*–*20*)	<*0.001*
RF positive, no. (%)	1331 (60)	121 (62)	0.7	282 (63)	0.8
Swollen joint count, median (IQR)	8 (5–12)	*9* (*6*–*13*)	0.05	*6* (*3*–*10*)	<*0.001*
Tender joint count, median (IQR)	14 (9–20)	*15* (*9*–*22*)	0.6	*12* (*7*–*20*)	*0.001*
ESR (mm/h), median (IQR)	24 (12–40)	26 (13–45)	0.4	25 (10–45)	0.4
CRP(mg/dl), median (IQR)	11 (5–26)	13 (5–34)	0.2	13 (5–34)	0.8
Global health VAS score, median (IQR)	*78* (*62*–*89*)	*72* (*55*–*85*)	*0.002*	72 (56–84)	0.8
DAS28, median (IQR)	6.0 (5.4–6.6)	*6.2* (*5.3*–*6.8*)	0.18	*5.7* (*5.0*–*6.4*)	<*0.001*
HAQ, median (IQR)	1.6 (1.0–2.1)	*1.6* (*1.1*–*2.1*)	0.9	*2.0* (*1.6*–*2.4*)	<*0.001*
Joint replacement surgery history, no. (%)	509 (21)	*40* (*19*)	0.4	*168* (*35*)	<*0.001*
Comorbidity status					
Any EARA^c^, no. (%)	431 (18)	*32* (*15*)	0.2	*148* (*30*)	<*0.001*
Pulmonary fibrosis, no. (%)	*35* (*2*)	*10* (*5*)	*0.001*	31 (6)	0.4
Any comorbidities^d^, no. (%)	1469 (61)	*135* (*62*)	0.7	*564* (*73*)	*0.003*
Hypertension history, no. (%)	685 (29)	*53* (*25*)	0.2	*279* (*37*)	*0.001*
Depression history, no. (%)	484 (21)	*42* (*20*)	0.8	*220* (*29*)	*0.007*
Diabetes history, no. (%)	173 (7)	22 (10)	0.1	63 (8)	0.3
Hyperlipidaemia history, no. (%)	402 (17)	38 (18)	0.7	178 (23)	0.09
Ischaemic heart disease history, no. (%)	133 (6)	17 (8)	0.1	57 (8)	0.8
Cancer history, no. (%)	*123* (*5*)	*20* (*9*)	*0.001*	69 (9)	0.8
Current and previous drug history					
Number of previous sDMARDs, median (IQR)	3 (2–3)	3 (2–3)	0.16	3 (2–4)	0.1
Current MTX, no. (%)	1566 (65)	136 (63)	0.5	496 (65)	0.6
Previous MTX, no. (%)	*2323* (*96*)	*203* (*94*)	*0.05*	737 (95)	0.3
Current steroids, no (%)	566 (23)	*39* (*18*)	0.07	*289* (*37*)	<*0.001*
After 1 bDMARD, no. (%)				217 (28)	
After 2 bDMARDs, no. (%)				321 (41)	
After 3 or more bDMARDs, no. (%)				237 (31)	

Statistically significant differences are given in italics

^a^Comparing between first-line TNFi users versus first-line TCZ users

^b^Comparing between first-line TCZ users versus subsequent-line TCZ users

^c^Extra-articular manifestations of rheumatoid arthritis (EARA)) including pulmonary fibrosis, sicca syndrome, serosal involvement (pleuritis/pericarditis), eye involvement, systemic vasculitis, nailfold vasculitis and other specified systemic features

^d^Comorbidities included one or more of high blood pressure, angina, heart attack, stroke, epilepsy, asthma, chronic bronchitis/emphysema, peptic ulcer, liver disease/hepatitis B or C, renal disease, tuberculosis, demyelination, diabetes, hyperthyroidism, depression or cancer

**Fig. 1:**
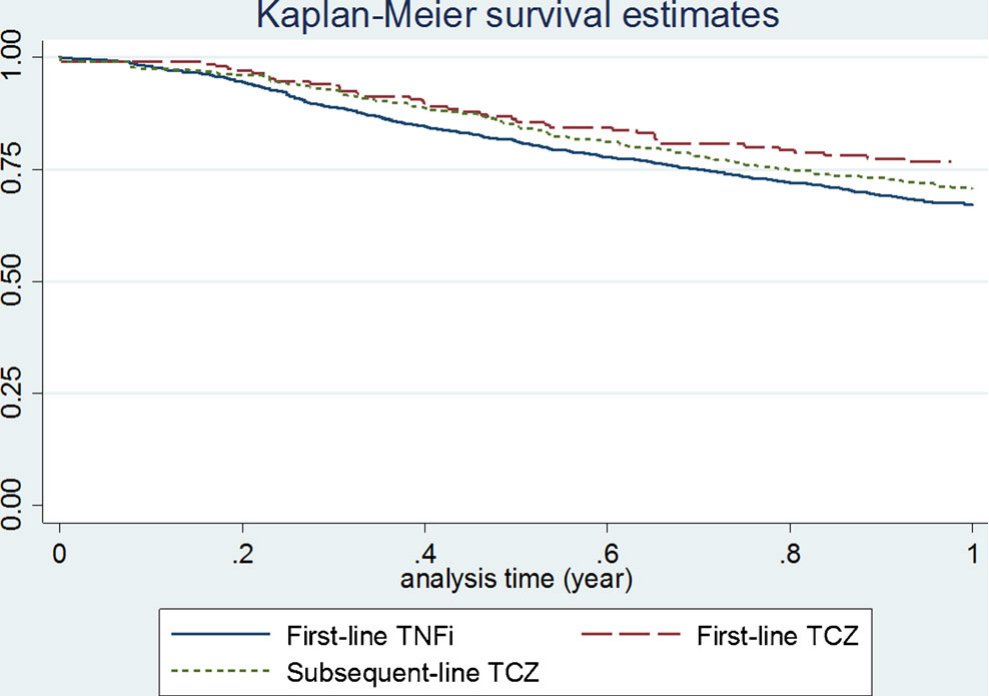
Drug survival curves of the first-line TNFi cohort, the first-line cohort and the subsequent-line TCZ cohort

Compared with the first-line TNFi cohort, the first-line TCZ cohort showed significantly lower DAS28 at 6 months (3.0 vs 3.5, *p* = 0.003) (Table 2) and higher absolute change in DAS28 (2.9 vs 2.4, *p* < 0.001). The percentage of good responders was slightly higher in the TCZ cohort than in the TNFi cohort (49 vs 42%); however, after adjustment using IPTW by propensity score, there was no difference in odds of achieving a higher EULAR response category. The percentage of patients who achieved DAS28 remission was higher in the first-line TCZ cohort compared to the TNFi cohort (42 vs 28%, *p* < 0.001), which remained significant after adjustment (adjusted odds ratio (OR) 1.86 (1.25–2.78)). However, an analysis of the individual components of DAS28 showed that the reduction in ESR was greater with TCZ (median ESR change 16.0 vs 7.0 mm/h, *p* < 0.001), but changes were similar in the other components of the DAS28 (online Supplementary Table 2). HAQ score and the proportion with MCID achievement at 6 months were also similar between the two cohorts (Table 2). Drug survival curves are shown in Fig. 1. The estimated 1-year survival was 0.67 (95% CI 0.65–0.69) for first-line TNFi users and was 0.77 (95% CI 0.70–0.82) for the first-line TCZ users. This difference was not statistically significant (*p* = 0.20).

**Table 2:** Changes in disease activity, disability and treatment response at 6 months following start of therapy

	First-line TNFi cohort (*N* = 2419)	First-line TCZ cohort (*N* = 217)	*P* value^a^	Subsequent-line TCZ cohort (*N* = 777)	*P* value^b^
DAS28 at month 6	(*N* = 1764)	(*N* = 158)	*0.003*	(*N* = 528)	*0.01*
Median (IQR)	*3.5* (*2.5*–*4.7*)	*3.0* (*1.8*–*4.6*)		*3.5* (*2.5*–*4.7*)	
EULAR response at month 6	(*N* = 1762)	(*N* = 158)	0.2	(*N* = 527)	*0.03*
Good response, no. (%)	737 (42)	77 (49)		*196* (*37*)	
Moderate response, no. (%)	665 (38)	52 (33)		*198* (*38*)	
No response, no. (%)	360 (20)	29 (18)		*133* (*25*)	
DAS28 remission at month 6	(*N* = 1764)	(*N* = 158)	<*0.001*	(*N* = 528)	*0.001*
No. (%)	*496* (*28*)	*66* (*42*)		*148* (*28*)	
OR for better EULAR response at month 6	(*N* = 2419)	(*N* = 217)		(*N* = 777)	
Unadjusted OR for better EULAR response (95% CI)	Referent	1.14 (0.85, 1.52)		*0.70* (*0.51*, *0.94*)	
		Referent			
Fully adjusted by IPTW OR for better EULAR response (95% CI)^c^	Referent	1.33 (0.92, 1.94)		0.73 (0.47, 1.15)	
		Referent			
OR for DAS28 remission at month 6	(*N* = 1627)	(*N* = 116)		(*N* = 589)	
Unadjusted OR for DAS28 remission (95% CI)	*Referent*	*1.62* (*1.19*, *2.22*)		*0.61* (*0.43*, *0.86*)	
		Referent			
Fully adjusted by IPTW OR for DAS28 remission (95% CI)^c^	Referent	*1.86* (*1.25*, *2.78*)		0.59 (0.34, 1.00)	
		Referent			
HAQ at month 6	(*N* = 1167)	(*N* = 100)	0.9	(*N* = 316)	<*0.001*
Median (IQR)	1.4 (0.6–1.9)	*1.3* (*0.8*–*1.9*)		*2.0* (*1.4*–*2.4*)	
Delta HAQ (baseline–month 6)	(*N* = 968)	(*N* = 88)	0.2	(*N* = 272)	*0.002*
Median (IQR)	0.1 (−0.1 to 0.5)	*0.3* (*−0.1* to *0.6*)		*0.0* (*−0.1*, *0.3*)	
MCID^d^	(*N* = 968)	(*N* = 88)	0.2	(*N* = 241)	<*0.001*
No. (%)	462 (48)	*49* (*46*)		*92* (*34*)	

Statistically significant differences are given in italics

^a^Comparing between first-line TNFi users versus first-line TCZ users

^b^Comparing between first-line TCZ users versus subsequent-line TCZ users

^c^Variables in propensity score for comparing between first-line TNFi users and TCZ users included age, gender, disease duration, BMI, DAS28 at baseline, HAQ score at baseline, concomitant use of MTX and steroids, current steroid, previous MTX use, pulmonary fibrosis presence, cancer history and any extra-articular manifestation presence. For comparing first-line TCZ users and subsequent-line TCZ users, any comorbidities’ presence was included instead of precious MTX use, pulmonary fibrosis presence and cancer history

^d^MCID was defined as a ≥0.22 decrease in HAQ score

### Comparison of baseline characteristics and treatment effectiveness between patients starting TCZ as a first-line versus subsequent-line biologic

Compared with the first-line cohort, the subsequent-line cohort had a significantly longer disease duration (12 vs 4 years, *p* < 0.001), slightly better disease activity (median DAS28 5.7 vs 6.2, *p* < 0.001) and lower functional capacity (median HAQ 2.0 vs 1.6, *p* < 0.001) (Table 1). There were higher proportions of any extra-articular manifestations (30 vs 15%, *p* < 0.001), any comorbidities (73 vs 62%, *P* = 0.003), reported depression (29 vs 20%, *p* = 0.001), hypertension (37 vs 25%, *p* = 0.003) and joint replacement surgery history (30 vs 15%, *P* < 0.001), but the proportions of pulmonary fibrosis were similar. Among subsequent-line TCZ cohort, most patients had received two or more prior bDMARDs (41% had two, 31% had three or more). Ninety-three percent had received at least one prior TNFi, and 55% had also received rituximab.

Compared to the first-line TCZ cohort, a lower proportion of the subsequent-line cohort achieved good EULAR responses and DAS28 remission (Table 2). However, after adjusting using IPTW by propensity score, there was no significant difference in the odds of achieving either better EULAR response or DAS28 remission between groups. In the subsequent-line cohort, change in HAQ score at 6 months was significantly smaller than in the first-line cohort (median delta HAQ 0.0 vs 0.3, *p* = 0.002) and a lower percentage of patients who achieved MCID (34 vs 49%, *p* < 0.001). Up to year 1, the drug retention rate for the subsequent-line TCZ users was estimated to be 0.71 (95% CI 0.67–0.74), which was similar to the first-line TCZ users (*p* = 0.23) (Fig. 1).

### Association between concurrent MTX use and response to TCZ

Within patients starting TCZ either as first or subsequent-line therapy, those who receive concomitant MTX therapy were younger and had fewer extra-articular manifestations and pulmonary fibrosis (online Supplementary Table 3).

**Fig. 2:**
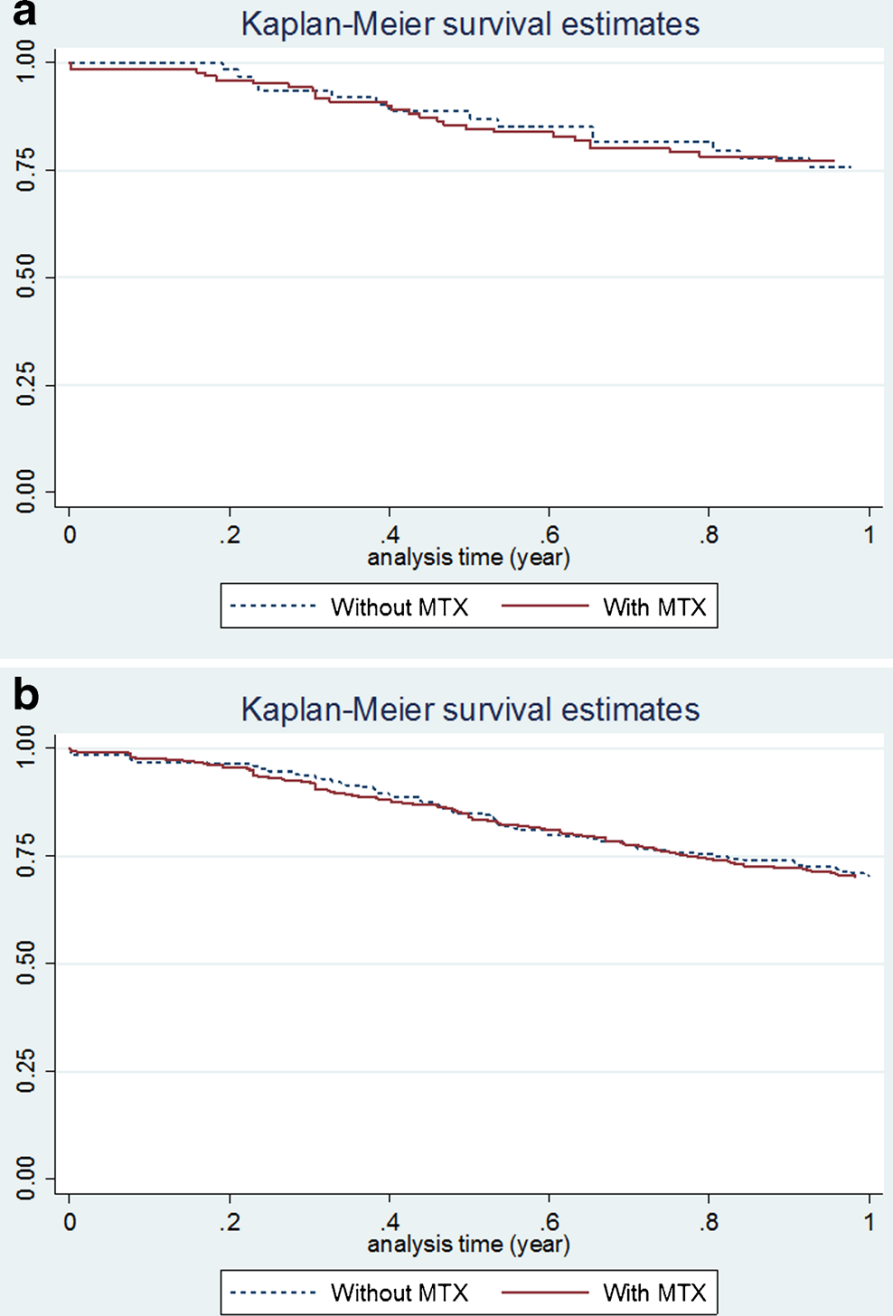
Drug survival curve comparison between the MTX users versus non-users. **a** Comparison among the first-line TCZ users. **b** Comparison among the subsequent-line TCZ users

After adjustment using propensity score, no significant difference in the proportion who met the EULAR response criteria or DAS28 remission was observed between MTX users and non-users, both in the first-line and subsequent-line cohorts, although low numbers in the first-line cohort resulted in wide confidence intervals (Table 3). At 6 months, functional activities were also similar between the cohorts. The drug survival curves were also similar between patients receiving TCZ with and without concomitant MTX therapy (Fig. 2a, b). The retention rates were estimated to be 0.77 (95% CI 0.68–0.84) for the first-line TCZ users with MTX and 0.76 (95% CI 0.62–0.85) for TCZ monotherapy (*p* = 0.10), 0.70 (95% CI 0.66–0.74) for the subsequent-line TCZ users with MTX and 0.70 (95% CI 0.64–0.76) for TCZ monotherapy (*p* = 0.50), respectively.

**Table 3:** Treatment response comparison between the MTX users versus non-users

	First-line TCZ users		*P* value^a^	Subsequent-line TCZ users		*P* value^b^
	With MTX (*N* = 136)	Without MTX (*N* = 81)		With MTX (*N* = 496)	Without MTX (*N* = 270)	
Disease activity						
DAS28 at baseline	(*N* = 136)	(*N* = 81)	*0.03*	(*N* = 496)	(*N* = 270)	0.4
Median (IQR)	*6.3* (*5.5*–*6.9*)	*5.8* (*5.2*–*6.6*)		5.7 (5.0–6.4)	5.7 (4.9–6.5)	
DAS28 at month 6	(*N* = 107)	(*N* = 51)	*0.04*	(*N* = 357)	(*N* = 166)	0.5
Median (IQR)	*2.9* (*1.6*–*4.3*)	*3.4* (*2.2*–*5.0*)		3.4 (2.5–4.6)	3.5 (2.4–5.0)	
EULAR response at month 6	(*N* = 107)	(*N* = 51)	0.2	(*N* = 356)	(*N* = 166)	0.8
Good response, no. (%)	57 (53)	20 (39)		135 (38)	59 (36)	
Moderate response, no. (%)	34 (32)	18 (35)		133 (37)	62 (7)	
No response, no. (%)	16 (15)	13 (26)		88 (25)	45 (27)	
DAS28 remission at month 6	(*N* = 107)	(*N* = 51)	*0.03*	(*N* = 357)	(*N* = 166)	0.7
No. (%)	*51* (*48*)	*15* (*29*)		98 (27)	48 (29)	
OR for better EULAR response at month 6	(*N* = 107)	(*N* = 51)		(*N* = 356)	(*N* = 166)	
Unadjusted OR for better EULAR response (95% CI)	1.48 (0.81, 2.71)	Referent		1.25 (0.90, 1.74)	Referent	
Fully adjusted by IPTW OR for better EULAR response (95% CI)^c^	1.58 (0.60, 4.17)	Referent		1.67 (0.99, 2.82)	Referent	
OR for DAS28 remission at month 6	(*N* = 107)	(*N* = 51)		(*N* = 357)	(*N* = 166)	
Unadjusted OR for DAS28 remission (95% CI)	1.78 (0.90, 3.52)	Referent		1.03 (0.72, 1.49)	Referent	
Fully adjusted by IPTW OR for DAS28 remission (95% CI)^c^	2.12 (0.69, 6.54)	Referent		1.31 (0.69, 2.49)	Referent	
Functional activity						
HAQ at month 6	(*N* = 67)	(*N* = 33)	0.4	(*N* = 213)	(*N* = 101)	0.3
Median (IQR)	1.4 (0.8–2.0)	1.1 (0.8, 1.8)		1.9 (1.4–2.3)	2.0 (1.4–2.4)	
Delta HAQ (baseline–month 6)	(*N* = 57)	(*N* = 31)	0.5	(*N* = 181)	(*N* = 89)	0.7
Median (IQR)	0.3 (−0.1 to 0.6)	0.4 (−0.1, 0.9)		0 (−0.1 to 0.3)	0.1 (−0.1 to 0.3)	
MCID^d^	(*N* = 57)	(*N* = 31)	0.2	(*N* = 181)	(*N* = 89)	0.9
No. (%)	29 (51)	20 (64)		61 (34)	31 (35)	

Statistically significant differences are given in italics

^a^Comparing between first-line TCZ users with MTX versus without it

^b^Comparing between subsequent-line TCZ users with MTX versus without it

^c^Variables in propensity score for first-line TCZ users included age, gender, disease duration, BMI, DAS28 at baseline, HAQ score at baseline, concomitant steroid use, previous MTX use, pulmonary fibrosis presence, ischaemic heart disease presence, any EARA presence and number of previous sDMARDs. For subsequent-line TCZ users, hypertension presence and depression presence were included instead of ischaemic heart disease presence

^d^MCID was defined as a ≥0.22 decrease in HAQ score

## Discussion

This is the first study both to describe the clinical characteristics of patients selected to receive TCZ as a first-line therapy in routine clinical use and to examine its effectiveness as a first-line and subsequent-line therapy. A majority of patients (78% of TCZ cohort) were starting this treatment as a subsequent-line biologic in the UK. In part, this may reflect the timing of UK approvals for use, which did not extend to first-line use until 2012, although even after this date, the majority of patients recruited were not starting TCZ as a first-line therapy.

Although similar with respect to disease history and disease activity, compared to patients starting TNFi as their first biologic, there were apparent differences in the comorbidity profile of patients starting TCZ, which may have influenced the choice of therapy. This was most noted with histories of pulmonary fibrosis and cancer. Although recent studies with long follow-up periods suggest that patients using TNFi are not at increased risk of interstitial pneumonia [25] or cancer development [26], several prior studies had indicated a possible link between TNFi usage and increasing risk of developing interstitial pneumonia and/or cancer [27–29] and both are included as relative contraindications to TNFi in current UK guidelines [29]. Despite the possible association between TCZ use and increases in fasting lipid levels found during clinical trials [30], we did not see any difference in baseline history of hyperlipidaemia or cardiovascular disease.

This analysis has suggested a superior effectiveness of TCZ compared to TNFi as a first bDMARD, particular in relation to the proportion of patients who achieved DAS28 remission. These results are similar to the ADACTA study (40% remission in the first-line TCZ users vs 11% in first-line ADA users) [6] and a retrospective study from Germany (44% in first-line TCZ users vs 30% in first-line TNFi users) [7]. A national registry in Portugal showed higher percentage of DAS remission in a first-line TCZ cohort (71% in first-line TCZ users vs 24% in first-line TNFi users, *p* < 0.001) [9]. However, it is possible that the significantly higher percentage of DAS28 remission achievement of TCZ users is explained through differences in the effects of each therapy in lowering the ESR and/or CRP, as when we compared the change in each DAS component between the first-line TCZ users and TNFi users, the improvements on the other components of DAS28 (SJC, TJC and patient VAS) were similar. A single-centre registry in Japan reported similar results in that patients who achieved DAS28 remission with TCZ showed lower mean ESR but, in fact, higher swollen joint counts [32]. Further studies focusing on other measures of inflammation besides the DAS28, such as improvements in synovitis as measured by ultrasound, are warranted. Whether this greater improvement in ESR is clinically important in terms of long-term clinical and radiographic outcomes, independent from improvements in swollen joint count, is not known. As another measure of effectiveness, we compared drug survivals of first-line TNFi and TCZ. Although there was a trend towards better survival with TCZ, this did not reach statistical significance. In addition, the analysis was not adjusted for baseline differences between patients because of the small quantity of data, and therefore, no conclusions can be drawn about the superiority of one drug over another in the longer term.

The majority of the patients in this study starting TCZ did so after one or more other biologic therapies, with many having failed >1. This will in part reflect the timing of introduction of different biologics into clinical practice. The ACT-LIFE study in routine clinical setting suggested that TCZ works better when used as a first-line therapy than as a subsequent line [11]. Our data also showed that the absolute proportion of patients achieving a good EULAR response among subsequent-line TCZ users was less than that seen among first-line users. However, after adjusting for differences in disease status at the start of the therapy, including disease duration and functional ability, these differences were no longer significant, perhaps indicating a group of patients with more resistant disease overall. These results are supported by those of a Japanese post-marketing surveillance which suggested that longer than 10-year disease duration and advanced disease (defined using Steinbrocker stage and class) were factors influencing lower treatment response at 28 weeks [10]. A further observational study also suggested that previous bDMARD exposure was not a significant predictor of treatment response of TCZ at month 6 [12], although there still remains a possibility that this difference did not reach statistical significance because of the small number of the first-line TCZ users.

In the UK, TCZ is also approved for use as monotherapy without MTX [5]. We did not see any difference in the influence of concurrent use of MTX on treatment response among the TCZ users, either as a first-line or subsequent-line biologic, which differs from that reported for TNFi [33]. While the evidence has suggested that TNFi may be more effective when used in combination with MTX, the observed benefits of concurrent MTX with TNFi are not well explained. In part, it may relate to factors associated with general patient drug tolerance and/or adherence. Studies also support a possible role for MTX in preventing anti-drug antibodies [34], although this area has been less studied with TCZ. In the current study, the sample size of the first-line TCZ users was very small, which may have prevented detection of smaller differences between MTX users and non-users. Whether the lack of benefit of MTX cotherapy with TCZ will still be apparent within larger datasets or over the longer term requires further study.

This study has a number of limitations. Firstly, the UK has restricted access to bDMARD therapy, demanding high disease activity (DAS28 >5.2). This may inflate the baseline assessments and affect clinical response, which may reduce the generalisability of the findings to other countries. However, the observed channelling of some patients towards treatments based on their comorbidity profile is less likely to differ between populations, and therefore, the treatment effectiveness data of this study could apply to patients with high disease activity in other countries.

Secondly, DAS28 was used as a primary treatment effectiveness measure. The DAS28 score might overestimate treatment effectiveness of TCZ due to the suppression of acute phase reactants, which may explain in part the greater lowering of ESR among patients receiving TCZ compared to TNFi. TCZ blocks IL-6 binding to its receptor, thereby preventing IL-6-mediated signal transduction, which alters the normalising of inflammatory markers (ESR and CRP) [35]. Several studies have reported that there is a discrepancy between DAS28 remission and Clinical Disease Activity Index (CDAI) remission (which does not include an acute-phase reactant component) [35–37]. Kawashiri et al. recommended that CDAI should be used as a disease activity measure for TCZ users [36]. However, the BSRBR-RA did not collect physician global assessment, a component of CDAI. Additionally, although DAS28-CRP tends to be lower than DAS-ESR and lower remission criteria are suggested [38], we were unable to distinguish DAS28-ESR and DAS28-CRP in the BSRBR-RA cohort, as the data were not always captured. However, in this study, other than DAS28, the drug survival curves and functional activity were also assessed as a measure of treatment effectiveness and did not identify any difference between treatments. Thirdly, this study might be affected by incomplete data. In approximately 20% of patients, the 6-month disease activity and HAQ data were missing, which is common in large observational longitudinal studies. To minimise response bias and attrition bias, multiple imputation model was used in this study. However, the effects of residual or unmeasured confounding factors could not be avoided. Although we compared the changes in disease activity and disability between therapies and therapy regimens, we did not compare the rates of adverse events. The ACT-RAY study did suggest a non-significant increase in the occurrence of raised liver enzymes among patients receiving TCZ in combination with MTX [14]. This study focused on short-term outcomes, but as further follow-up is accrued, future studies should look at the longer-term tolerability and maintenance of effect, including the risk of adverse events. It would also be interesting to assess the effectiveness of subsequent bDMARDs after primary failure with TCZ therapy. Although a recent retrospective study suggested that TNFi could be superior than abatacept for the patients showing inadequate response to TCZ [39], there has been sparse evidence in the bDMARD selection after TCZ. Our data suggest that any study of comparative drug safety or long-term tolerability must also take into account differences in the proportions of baseline comorbidities, particularly pulmonary disease.

In conclusion, this national cohort study of real-world TCZ use has shown that with an expanding choice of therapies for patients who are sDMARD inadequate responders, similar improvements in disease activity and disability are seen between patients starting either TCZ or TNFi, with the exception of a greater improvement in ESR among TCZ users. The proportion of responders among patients who start TCZ following an inadequate response to one or more previous biologic therapies is lower compared to first-line use but may largely be explained by differences in disease severity. The benefit of MTX cotherapy with TCZ was not apparent in this study; however, this may be underestimated because of the very small sample size of the first-line TCZ cohort.

## Electronic supplementary material

Table 1. Number and percentage of missing values in the variables (DOCX 14 kb)

Table 2. Absolute change in DAS28 and DAS28 components (DOCX 11 kb)

Table 3. Baseline characteristics comparison between the MTX users versus non-users (DOCX 19 kb)

## References

[1] Choy EHS, Panayi GS (2001). Cytokine pathways and joint inflammation in rheumatoid arthritis. N Engl J Med.

[2] Smolen JS, Landewe R, Breedveld FC, Buch M, Burmester G, Dougados M, Emery P, Gaujoux-Viala C, Gossec L, Nam J, Ramiro S, Winthrop K, de Wit M, Aletaha D, Betteridge N, Bijlsma JW, Boers M, Buttgereit F, Combe B, Cutolo M, Damjanov N, Hazes JM, Kouloumas M, Kvien TK, Mariette X, Pavelka K, van Riel PL, Rubbert-Roth A, Scholte-Voshaar M, Scott DL, Sokka-Isler T, Wong JB, van der Heijde D (2014). EULAR recommendations for the management of rheumatoid arthritis with synthetic and biological disease-modifying antirheumatic drugs: 2013 update. Ann Rheum Dis.

[3] Nishimoto N, Yoshizaki K, Maeda K, Kuritani T, Deguchi H, Sato B, Imai N, Suemura M, Kakehi T, Tagaki N, Kishimoto T (2003). Toxicity, pharmacokinetics, and dose-finding study of repetitive treatment with. J Rheumatol.

[4] NICE (2010) Tocilizumab for the treatment of rheumatoid arthritis. NICE Technol. Apprais. Guid. 198

[5] NICE (2012) Tocilizumab for the treatment of rheumatoid arthritis (rapid review of technology appraisal guidance 198). In: NICE Technol. Apprais. Guid. 247. https://www.nice.org.uk/guidance/ta247

[6] Gabay C, Emery P, van Vollenhoven R, Dikranian A, Alten R, Pavelka K, Klearman M, Musselman D, Agarwal S, Green J, Kavanaugh A (2013). Tocilizumab monotherapy versus adalimumab monotherapy for treatment of rheumatoid arthritis (ADACTA): a randomised, double-blind, controlled phase 4 trial. Lancet.

[7] Backhaus M, Kaufmann J, Richter C, Wassenberg S, Roske A-E, Hellmann P, Gaubitz M (2015). Comparison of tocilizumab and tumour necrosis factor inhibitors in rheumatoid arthritis: a retrospective analysis of 1603 patients managed in routine clinical practice. Clin Rheumatol.

[8] Kaufmann J, Feist E, Roske AE, Schmidt W a (2013). Monotherapy with tocilizumab or TNF-alpha inhibitors in patients with rheumatoid arthritis: efficacy, treatment satisfaction, and persistence in routine clinical practice. Clin Rheumatol.

[9] Romão VC, Santos MJ, Polido-Pereira J, Duarte C, Nero P, Miguel C, Costa JA, Bernardes M, Pimentel-Santos FM, Barcelos F, Costa L, António J, Gomes M, Alberto J, Branco JC, Canas J, António J, Fonseca JE, Canhão H (2015) Comparative effectiveness of tocilizumab and TNF inhibitors in rheumatoid arthritis patients: data from the Rheumatic Diseases Portuguese Register, Reuma. pt. 2015 10.1155/2015/279890 PMC4427085 26000286

[10] Koike T, Harigai M, Inokuma S, Ishiguro N, Ryu J, Takeuchi T, Takei S, Tanaka Y, Sano Y, Yaguramaki H, Yamanaka H (2014). Effectiveness and safety of tocilizumab: postmarketing surveillance of 7901 patients with rheumatoid arthritis in Japan. J Rheumatol.

[11] Balsa A, Tovar Beltran JV, Caliz Caliz R, Mateo Bernardo I, Garcia-Vicun AR, Rodriguez-Gomez M, Belmonte Serrano MA, Marras C, Loza Cortina E, Perez-Pampin E, Vila V (2015). Patterns of use and dosing of tocilizumab in the treatment of patients with rheumatoid arthritis in routine clinical practice: the ACT-LIFE study. Rheumatol Int.

[12] Narváez J, Magallares B, Díaz Torné C, Hernández MV, Reina D, Corominas H, Sanmartí R, LLobet JM, Rodriguez de la Serna A, Nolla JM (2015). Predictive factors for induction of remission in patients with active rheumatoid arthritis treated with tocilizumab in clinical practice. Semin Arthritis Rheum.

[13] Dougados M, Kissel K, Conaghan PG, Mola EM, Schett G, Gerli R, Hansen MS, Amital H, Xavier RM, Troum O, Bernasconi C, Huizinga TWJ (2014). Clinical, radiographic and immunogenic effects after 1 year of tocilizumab-based treatment strategies in rheumatoid arthritis: the ACT-RAY study. Ann Rheum Dis.

[14] Huizinga TWJ, Conaghan PG, Martin-Mola E, Schett G, Amital H, Xavier RM, Troum O, Aassi M, Bernasconi C, Dougados M (2015). Clinical and radiographic outcomes at 2 years and the effect of tocilizumab discontinuation following sustained remission in the second and third year of the ACT-RAY study. Ann Rheum Dis.

[15] Kaneko Y, Atsumi T, Tanaka Y, Inoo M, Kobayashi-Haraoka H, Amano K, Miyata M, Murakawa Y, Yasuoka H, Hirata S, Nagasawa H, Tanaka E, Miyasaka N, Yamanaka H, Yamamoto K (2016) Comparison of adding tocilizumab to methotrexate with switching to tocilizumab in patients with rheumatoid arthritis with inadequate response to methotrexate: 52-week results from a prospective, randomised, controlled study (SURPRISE study ). 1–7. doi: 10.1136/annrheumdis-2015-208426 10.1136/annrheumdis-2015-208426 PMC5099201 26733110

[16] Kojima T, Yabe Y, Kaneko A, Takahashi N, Funahashi K, Kato D, Hanabayashi M, Asai S, Hirabara S, Asai N, Hirano Y, Hayashi M, Miyake H, Kojima M, Ishiguro N (2015). Importance of methotrexate therapy concomitant with tocilizumab treatment in achieving better clinical outcomes for rheumatoid arthritis patients with high disease activity: an observational cohort study. Rheumatology (Oxford).

[17] Gabay C, Riek M, Hetland ML, Hauge E-M, Pavelka K, Tomšič M, Canhao H, Chatzdionysiou K, Lukina G, Nordström DC, Lie E, Ancuta I, Hernández MV, van Riel PLMC, van Vollenhoven R, Kvien TK (2015). Effectiveness of tocilizumab with and without synthetic disease-modifying antirheumatic drugs in rheumatoid arthritis: results from a European collaborative study. Ann Rheum Dis annrheumdis.

[18] Izumi K, Kaneko Y, Yasuoka H, Seta N, Kameda H, Kuwana M, Takeuchi T (2015). Tocilizumab is clinically, functionally, and radiographically effective and safe either with or without low-dose methotrexate in active rheumatoid arthritis patients with inadequate responses to DMARDs and/or TNF inhibitors: a single-center retrospective. Mod Rheumatol.

[19] Bruce B, Fries JF (2005). The Health Assessment Questionnaire (HAQ). Clin Exp Rheumatol.

[20] van Gestel a M, Prevoo ML, van’t Hof M a, van Rijswijk MH, van de Putte LB, van Riel PL (1996). Development and validation of the European League Against Rheumatism response criteria for rheumatoid arthritis. Comparison with the preliminary American College of Rheumatology and the World Health Organization/International League Against Rheumatism Criteria. Arthritis Rheum.

[21] van Riel PL, van Gestel a M (2000). Clinical outcome measures in rheumatoid arthritis. Ann Rheum Dis.

[22] Beaton DE, Bombardier C, Katz JN, Wright JG, Wells G, Boers M, Strand V, Shea B (2001). Looking for important change/differences in studies of responsiveness. J Rheumatol.

[23] Fransen J, Stucki G, van Riel PLCM (2003). Rheumatoid arthritis measures: Disease Activity Score (DAS), Disease Activity Score-28 (DAS28), Rapid Assessment of Disease Activity in Rheumatology (RADAR), and Rheumatoid Arthritis Disease Activity Index (RADAI). Arthritis Rheum.

[24] Cole SR, Hernan MA (2008). Constructing inverse probability weights for marginal structural models. Am J Epidemiol.

[25] Ramos-Casals M, Perez-Alvarez R, Perez-De-Lis M, Xaubet A, Bosch X (2011). Pulmonary disorders induced by monoclonal antibodies in patients with rheumatologic autoimmune diseases. Am J Med.

[26] Mercer LK, Lunt M, Low ALS, Dixon WG, Watson KD, Symmons DPM, Hyrich KL (2014) Risk of solid cancer in patients exposed to anti-tumour necrosis factor therapy: results from the British Society for Rheumatology Biologics Register for Rheumatoid Arthritis. Ann Rheum Dis:1–7. doi:10.1136/annrheumdis-2013-204851 10.1136/annrheumdis-2013-204851 PMC4431340 24685910

[27] Khasnis AA, Calabrese LH (2010). Tumor necrosis factor inhibitors and lung disease: a paradox of efficacy and risk. Semin Arthritis Rheum.

[28] Bongartz T, Sutton AJ, Sweeting MJ, Buchan I, Matteson EL, Montori V (2006). Anti-TNF antibody therapy in rheumatoid arthritis and the risk of serious infections and malignancies: systematic review and meta-analysis of rare harmful effects in randomized controlled trials. JAMA.

[29] Ding T, Ledingham J, Luqmani R, Westlake S, Hyrich K, Lunt M, Kiely P, Bukhari M, Abernethy R, Bosworth A, Ostor A, Gadsby K, McKenna F, Finney D, Dixey J, Deighton C (2010). BSR and BHPR rheumatoid arthritis guidelines on safety of anti-TNF therapies. Rheumatology (Oxford).

[30] Schiff MH, Kremer JM, Jahreis A, Vernon E, Isaacs JD, van Vollenhoven RF (2011). Integrated safety in tocilizumab clinical trials. Arthritis Res Ther.

[31] Akiyama M, Kaneko Y, Yamaoka K, Kondo H, Takeuchi T (2016). Association of disease activity with acute exacerbation of interstitial lung disease during tocilizumab treatment in patients with rheumatoid arthritis: a retrospective, case???Control study. Rheumatol Int.

[32] Yoshida K, Tokuda Y, Oshikawa H, Utsunomiya M, Kobayashi T, Kimura M, Deshpande G a, Matsui K, Kishimoto M (2011). An observational study of tocilizumab and TNF-α inhibitor use in a Japanese community hospital: different remission rates, similar drug survival and safety. Rheumatology (Oxford).

[33] Hyrich KL, Symmons DPM, Watson KD, Silman AJ (2006). Comparison of the response to infliximab or etanercept monotherapy with the response to cotherapy with methotrexate or another disease-modifying antirheumatic drug in patients with rheumatoid arthritis: results from the British Society for Rheumatology Biologics Register. Arthritis Rheum.

[34] Jani M, Chinoy H, Warren RB, Griffiths CEM, Plant D, Fu B, Morgan AW, Wilson AG, Isaacs JD, Hyrich K, Barton A (2015). Clinical utility of random anti-tumor necrosis factor drug-level testing and measurement of antidrug antibodies on the long-term treatment response in rheumatoid arthritis. Arthritis Rheumatol.

[35] Smolen JS, Aletaha D (2011). Interleukin-6 receptor inhibition with tocilizumab and attainment of disease remission in rheumatoid arthritis: the role of acute-phase reactants. Arthritis Rheum.

[36] Kawashiri S-Y, Kawakami A, Iwamoto N, Fujikawa K, Aramaki T, Tamai M, Yamasaki S, Nakamura H, Ueki Y, Migita K, Mizokami A, Origuchi T, Aoyagi K, Eguchi K (2011). Disease activity score 28 may overestimate the remission induction of rheumatoid arthritis patients treated with tocilizumab: comparison with the remission by the clinical disease activity index. Mod Rheumatol.

[37] Funahashi K, Koyano S, Miura T, Hagiwara T, Okuda K, Matsubara T (2009). Efficacy of tocilizumab and evaluation of clinical remission as determined by CDAI and MMP-3 level. Mod Rheumatol.

[38] Inoue E, Yamanaka H, Hara M, Tomatsu T, Kamatani N (2007). Comparison of Disease Activity Score (DAS)28- erythrocyte sedimentation rate and DAS28- C-reactive protein threshold values. Ann Rheum Dis.

[39] Akiyama M, Kaneko Y, Kondo H, Takeuchi T (2016) Comparison of the clinical effectiveness of tumour necrosis factor inhibitors and abatacept after insufficient response to tocilizumab in patients with rheumatoid arthritis. Clin Rheumatol:1–6. doi:10.1007/s10067-016-3227-8 10.1007/s10067-016-3227-8 26971256

