# Supplementary material for: Use and effectiveness of tocilizumab among patients with rheumatoid arthritis: an observational study from the British Society for Rheumatology Biologics Register for rheumatoid arthritis
Source: Clin Rheumatol. 2016 Dec 2;36(2):241–50. doi: 10.1007/s10067-016-3485-5 (PMC5290047; doi:10.1007/s10067-016-3485-5)
Supplement: Supplementary file 1 — Number and percentage of missing values in the variables (DOCX 14 kb) [file 10067_2016_3485_MOESM1_ESM.docx]

**Online supplementary Table 1. Number and percentage of missing values in the variables**

| **Missing values, no. (%)** | **First-line TNFi cohort (N=2419)** | **First-line TCZ cohort (N=217)** | **Subsequent-line TCZ cohort (N=777)** |
| --- | --- | --- | --- |
| **Baseline information** | | | |
| Age | 0 (0) | 0 (0) | 0 (0) |
| Sex | 0 (0) | 0 (0) | 0 (0) |
| Smoking status | 89 (4) | 8 (4) | 283 (36) |
| BMI | 613 (25) | 66 (30) | 380 (49) |
| Disease duration | 59 (2) | 5 (2) | 14 (2) |
| RF | 216 (9) | 22 (10) | 330 (42) |
| Swollen joint count | 74 (3) | 6 (3) | 30 (4) |
| Tender joint count | 70 (3) | 5 (2) | 31 (4) |
| ESR | 645 (27) | 48 (22) | 229 (29) |
| CRP | 1092 (45) | 115 (53) | 342 (44) |
| Global health VAS score | 127 (5) | 9 (4) | 50 (6) |
| DAS28 at baseline | 2 (1) | 0 (0) | 2 (0.3) |
| HAQ score at baseline | 618 (26) | 60 (28) | 187 (24) |
| Joint replacement surgery history | 23 (1) | 3 (1) | 298 (38) |
| Presence of any extra-articular manifestations | 64 (3) | 3 (1) | 286 (37) |
| Presence of pulmonary fibrosis | 87 (4) | 7 (3) | 295 (38) |
| Any comorbidities | 0 (0) | 0 (0) | 0 (0) |
| Hypertension history | 61 (3) | 4 (1) | 20 (3) |
| Depression history | 85 (4) | 6 (3) | 26 (3) |
| Diabetes history | 23 (1) | 3 (1) | 10 (1) |
| Hyperlipidaemia history | 0 (0) | 0 (0) | 0 (0) |
| Ischaemic heart disease history | 40 (2) | 5 (2) | 18 (2) |
| Cancer history | 0 (0) | 0 (0) | 0 (0) |
| Number of previous sDMARDs | 0 (0) | 0 (0) | 3 (1) |
| Current MTX | 0 (0) | 0 (0) | 11 (1) |
| Previous MTX | 0 (0) | 0 (0) | 3 (1) |
| Current steroids | 0 (0) | 0 (0) | 0 (0) |
| Number of previous bDMARDs | 6 (0.3) | 0 (0) | 2 (0.3) |
| **Follow-up data** | | | |
| DAS28 at month 6 | 655 (27) | 59 (27) | 249 (32) |
| HAQ score at month 6 | 1252 (52) | 117 (54) | 461 (59) |
| Drug survival by Year 1 | 728 (30) | 67 (31) | 197 (25) |

ESR at baseline was not available in 26% of the cohorts, where the DAS28-CRP was calculated instead of DAS28-ESR. Missing data were imputed in regression models using multiple imputation.
