# Supplementary material for: Use and effectiveness of tocilizumab among patients with rheumatoid arthritis: an observational study from the British Society for Rheumatology Biologics Register for rheumatoid arthritis
Source: Clin Rheumatol. 2016 Dec 2;36(2):241–50. doi: 10.1007/s10067-016-3485-5 (PMC5290047; doi:10.1007/s10067-016-3485-5)
Supplement: Supplementary file 2 — Absolute change in DAS28 and DAS28 components (DOCX 11 kb) [file 10067_2016_3485_MOESM2_ESM.docx]

**Online supplementary Table 2. Absolute change in DAS28 and DAS28 components**

|  | | **First-line TNFi cohort　(N=2419)** | **First-line TCZ cohort　(N=217)** | **P-value** |
| --- | --- | --- | --- | --- |
| **Delta DAS28 (baseline-month 6)** | | (N=1762) | (N=158) |  |
|  | Median (IQR) | **2.4 (1.2-3.4)** | **2.9 (1.4-4.4)** | **0.0004** |
| **Change in DAS28 component** | |  |  |  |
| Delta SJC28 (baseline-month6) | | (N=1735) | (N=159) |  |
|  | Median (IQR) | 5 (2-9) | 6 (3-10) | 0.2 |
| Delta TJC28 (baseline-month6) | | (N=1739) | (N=160) |  |
|  | Median (IQR) | 9 (4-15) | 9 (4-16) | 0.7 |
| Delta ESR (baseline-month6) | | (N=1191) | (N=116) |  |
|  | Median (IQR) | **7.0 (0.0-19.0)** | **16.0 (5.0-35.5)** | **<0.001** |
| Delta CRP (baseline-month6) | | (N=858) | (N=68) |  |
|  | Median (IQR) | 4.0 (0.0-16.0) | 7.5 (0.1-21.1) | 0.2 |
| Delta global health VAS score (baseline-month6) | | (N=1464) | (N=129) |  |
|  | Median (IQR) | 35 (15-55) | 29 (15-50) | 0.09 |

Comparing between first-line TCZ users and TNFi users. Statistically significant differences are given in bold.
