# Supplementary material for: Use and effectiveness of tocilizumab among patients with rheumatoid arthritis: an observational study from the British Society for Rheumatology Biologics Register for rheumatoid arthritis
Source: Clin Rheumatol. 2016 Dec 2;36(2):241–50. doi: 10.1007/s10067-016-3485-5 (PMC5290047; doi:10.1007/s10067-016-3485-5)
Supplement: Supplementary file 3 — Baseline characteristics comparison between the MTX users versus non-users (DOCX 19 kb) [file 10067_2016_3485_MOESM3_ESM.docx]

**Online supplementary Table 3. Baseline characteristics comparison between the MTX users versus non-users**

|  |  | First-line TCZ cohort | | P-value ^a^ | Subsequent-line TCZ cohort | | P-value ^b^ |
| --- | --- | --- | --- | --- | --- | --- | --- |
|  |  | With MTX (n=136) | Without MTX (n=81) |  | With MTX (n=496) | Without MTX (n=270) |  |
| **Demographics** | | | | | | | |
|  | Age, median (IQR) | **56 (48-66)** | **61 (53-8)** | **0.01** | **57 (48-64)** | **62 (53-68)** | **<0.0001** |
|  | Female, no. (%) | 102 (75) | 57 (70) | 0.5 | 402 (81) | 207 (77) | 0.2 |
|  | Current smokers, no. (%) | 30 (22) | 11 (15) | 0.2 | 57 (19) | 35 (19) | 0.9 |
|  | BMI, median (IQR) | 28.9 (25.9-33.4) | 26.5 (24.2-32.0) | 0.06 | **28.4 (24.4-33.7)** | **26.6 (23.2-30.4)** | **0.0006** |
| **Disease characteristics** | | | | | | | |
|  | Disease duration (years), median (IQR) | 4 (2-10) | 5 (2-13) | 0.9 | 11 (6-19) | 12 (6-21) | 0.6 |
|  | RF positive, no. (%) | 75 (60) | 46 (67) | 0.3 | 166 (62) | 111 (65) | 0.5 |
|  | Swollen joint count, median (IQR) | 10 (6-14) | 8 (5-12) | 0.1 | 6 (4-9) | 6 (3-10) | 0.9 |
|  | Tender joint count, median (IQR) | 15 (10-22) | 13 (8-21) | 0.06 | 12 (7-20) | 11 (7-18) | 0.08 |
|  | ESR (mm/hour), median (IQR) | 27 (13-41) | 25 (12-46) | 0.7 | **24 (9-40)** | **29 (11-56)** | **0.03** |
|  | CRP(mg/dl), median (IQR) | 11 (5-32) | 22 (5-35) | 0.6 | 12 (5-35) | 15 (5-36) | 0.4 |
|  | Global health VAS score, median (IQR) | 74 (60-85) | 70 (50-90) | 0.6 | 72 (57-80) | 71 (56-85) | 0.9 |
|  | DAS28, median (IQR) | **6.3 (5.5-6.9)** | **5.8 (5.2-6.6)** | **0.03** | 5.7 (5.0-6.4) | 5.7 (4.9-6.5) | 0.4 |
|  | HAQ, median (IQR) | 1.7 (1.1-2.1) | 1.6 (1.0-2.1) | 0.5 | 2 (1.6-2.4) | 2 (1.6-2.5) | 0.3 |
|  | Joint replacement surgery history, no. (%) | 24 (18) | 16 (20) | 0.7 | 95 (33) | 71 (38) | 0.3 |
| **Comorbidity status** | | | | | | | |
|  | Any extra-articular manifestations ^c^, no. (%) | **14 (10)** | **18 (23)** | **0.02** | **81 (27)** | **66 (25)** | **0.05** |
|  | Pulmonary fibrosis, no. (%) | **3 (2)** | **7 (9)** | **0.03** | **9 (3)** | **28 (12)** | **<0.0001** |
|  | Any comorbidities ^d^, no. (%) | 79 (58) | 56 (69) | 0.1 | 354 (71) | 202 (75) | 0.3 |
|  | Hypertension history, no. (%) | 31 (23) | 22 (28) | 0.4 | **166 (34)** | **110 (42)** | **0.04** |
|  | Depression history, no. (%) | 26 (19) | 16 (21) | 0.8 | **155 (32)** | **64 (25)** | **0.03** |
|  | Diabetes history, no. (%) | 16 (12) | 6 (8) | 0.3 | 41 (8) | 21 (8) | 0.8 |
|  | Hyperlipidaemia history, no. (%) | 24 (18) | 14 (17) | 0.9 | 112 (23) | 64 (24) | 0.7 |
|  | Ischaemic heart disease history, no. (%) | 14 (10) | 3 (4) | 0.09 | 36 (7) | 20 (7) | 1 |
|  | Cancer history, no. (%) | 11 (8) | 9 (11) | 0.5 | 46 (9) | 23 (9) | 0.7 |
| **Current and previous drug history** | | | | | | | |
|  | Number of previous sDMARDs, median (IQR) | 3 (2-3) | 3 (2-3) | 0.09 | **3 (2-3)** | **3 (2-4)** | **<0.0001** |
|  | Previous MTX, no. (%) | **134 (99)** | **69 (85)** | **<0.0001** | **491 (99)** | **240 (89)** | **<0.0001** |
|  | Current steroids, no (%) | 20 (15) | 19 (23) | 0.1 | 171 (34) | 112 (41) | 0.06 |
|  | After 1 bDMARD, no. (%) |  | | | **111 (22)** | **102 (38)** | **<0.0001** |
|  | After 2 bDMARDs, no. (%) |  |  |  | **207 (42)** | **110 (41)** |  |
|  | After 3 or more bDMARDs, no. (%) |  |  |  | **177 (36)** | **57 (21)** |  |

Comparing between ^a^ first-line TCZ users with MTX versus without it and ^b^ subsequent-line TCZ users with MTX versus without it. Statistically significant differences are given in bold. These were used to estimate propensity score. ^c^ Extra-articular manifestations including pulmonary fibrosis, sicca syndrome, serosal involvement (pleuritis/pericarditis), eye involvement, systemic vasculitis, nailfold vasculitis and other specified systemic features. ^d^ Comorbidities included one or more of high blood pressure, angina, heart attack, stroke, epilepsy, asthma, chronic bronchitis/emphysema, peptic ulcer, liver disease/hepatitis B or C, renal disease, tuberculosis, demyelination, diabetes, hyperthyroidism, depression or cancer.
